# Mechanism of Hypercholesterolemia-Induced Atherosclerosis

**DOI:** 10.31083/j.rcm2306212

**Published:** 2022-06-09

**Authors:** Kailash Prasad, Manish Mishra

**Affiliations:** ^1^Department of Physiology (APP), College of Medicine, University of Saskatchewan, Saskatoon, SK S7N 5A2, Canada; ^2^Department of Pharmacology, Dalhousie University, Halifax, NS B3H 4R2, Canada

**Keywords:** hypercholesterolemia, reactive oxygen species, atherosclerosis, cell adhesion molecules, cytokines, advanced glycation end products, C-reactive protein, nuclear factor-kappa B, atherogenic biomolecules

## Abstract

Hypercholesterolemia is involved in the development of atherosclerosis and is a risk factor for coronary artery disease, stroke, and peripheral vascular disease. This paper deals with the mechanism of development of hypercholesterolemic atherosclerosis. Hypercholesterolemia increases the formation of numerous atherogenic biomolecules including reactive oxygen species (ROS), proinflammatory cytokines [interleukin (IL)-1, IL-2, IL-6, IL-8, tumor necrosis factor-alpha (TNF-α)], expression of intercellular adhesion molecule-1 (ICAM-1), vascular cell adhesion molecule-1 (VCAM-1), E-selectin, monocyte chemoattractant protein-1 (MCP-1), granulocyte macrophage-colony stimulating factor (GM-CSF) and numerous growth factors [insulin-like growth factor-1 (IGF-1), platelet-derived growth factor-1 (PDGF-1) and transforming growth factor-beta (TGF-β)]. ROS mildly oxidizes low-density lipoprotein-cholesterol (LDL-C) to form minimally modified LDL (MM-LDL) which is further oxidized to form oxidized LDL (OX-LDL). Hypercholesterolemia also activates nuclear factor-kappa-B (NF-κB). The above atherogenic biomolecules are involved in the development of atherosclerosis which has been described in detail. Hypercholesterolemia also assists in the development of atherosclerosis through AGE (advanced glycation end-products)-RAGE (receptor for AGE) axis and C-reactive protein (CRP). Hypercholesterolemia is associated with increases in AGE, oxidative stress [AGE/sRAGE (soluble receptor for AGE)] and C-reactive protein, and decreases in the sRAGE, which are known to be implicated in the development of atherosclerosis. In conclusion, hypercholesterolemia induces atherosclerosis through increases in atherogenic biomolecules, AGE-RAGE axis and CRP.

## 1. Introduction

Atherosclerosis affects medium and large-sized arteries and is characterized by focal thickening of the intima of the arteries and deposition of lipid, resulting in narrowing of the arteries. Atherosclerosis leads to cardiovascular diseases [1]. There are numerous factors including hyperlipidemia [2, 3], diabetes [4], hypertension, cigarette smoking [5], obesity [6], hyperhomocysteinemia [7], and elevated serum C-reactive protein [8, 9] which are involved in the development of atherosclerosis. The term hyperlipidemia refers to increased levels of serum total cholesterol (TC), low-density lipoprotein-cholesterol (LDL-C) and triglycerides (TG), or a combination of all the three. A major risk factor for coronary artery disease is hyperlipidemia [3, 10]. CAD (coronary artery disease) risk increases by 2% to 3% for every 1% increase in serum cholesterol [11]. A 10% reduction of serum cholesterol reduces the risk of CAD by half for men of 40 yrs of age and by 25% for men 60 yrs of age over 5 yrs [11]. An increase of 10 mg/dL of LDL-C was associated with a 12% increase in the risk of cardiovascular disease (CVD) [12]. The serum TG levels are strongly associated with CAD [13, 14]. There is a strong inverse correlation of high-density lipoprotein cholesterol (HDL-C) with atherosclerotic CAD. High serum HDL-C levels reduce the rate of atherogenesis [15], while low levels of HDL-C accelerate atherosclerosis [16]. The risk of CAD is increased by 2% to 3% for every 1 mg/dL reduction in the levels of HDL-C [17]. The ratio of TC/HDL-C >3.5 in men and >4.5 in women, while the ratio of LDL-C/ HDL-C >3.5 in men, and >3.0 in women are risk of cardiovascular diseases [18]. Reactive oxygen species (ROS) [19, 20, 21, 22], and advanced glycation end products (AGE) and its cell receptor RAGE (receptor for AGE) and soluble receptor for AGE (sRAGE) [23, 24] have been implicated in the development of atherosclerosis. AGE and its cell receptors, sRAGE and esRAGE (endogenous secretory receptor for AGE) have been implicated in various diseases including non-ST segment elevated myocardial infarction (NSTEMI) [25], restenosis following PCI (percutaneous coronary intervention) [26] and accelerated atherosclerosis with streptozotocin-induced diabetes in apo-E-deficient mice [27]. This paper deals with the mechanism of hypercholesterolemia-induced atherosclerosis, with special reference to ROS and AGE-RAGE axis and C-reactive protein (CRP).

## 2. Effects of Hypercholesterolemia on Atherogenic Biomolecules

**Fig. 1.**
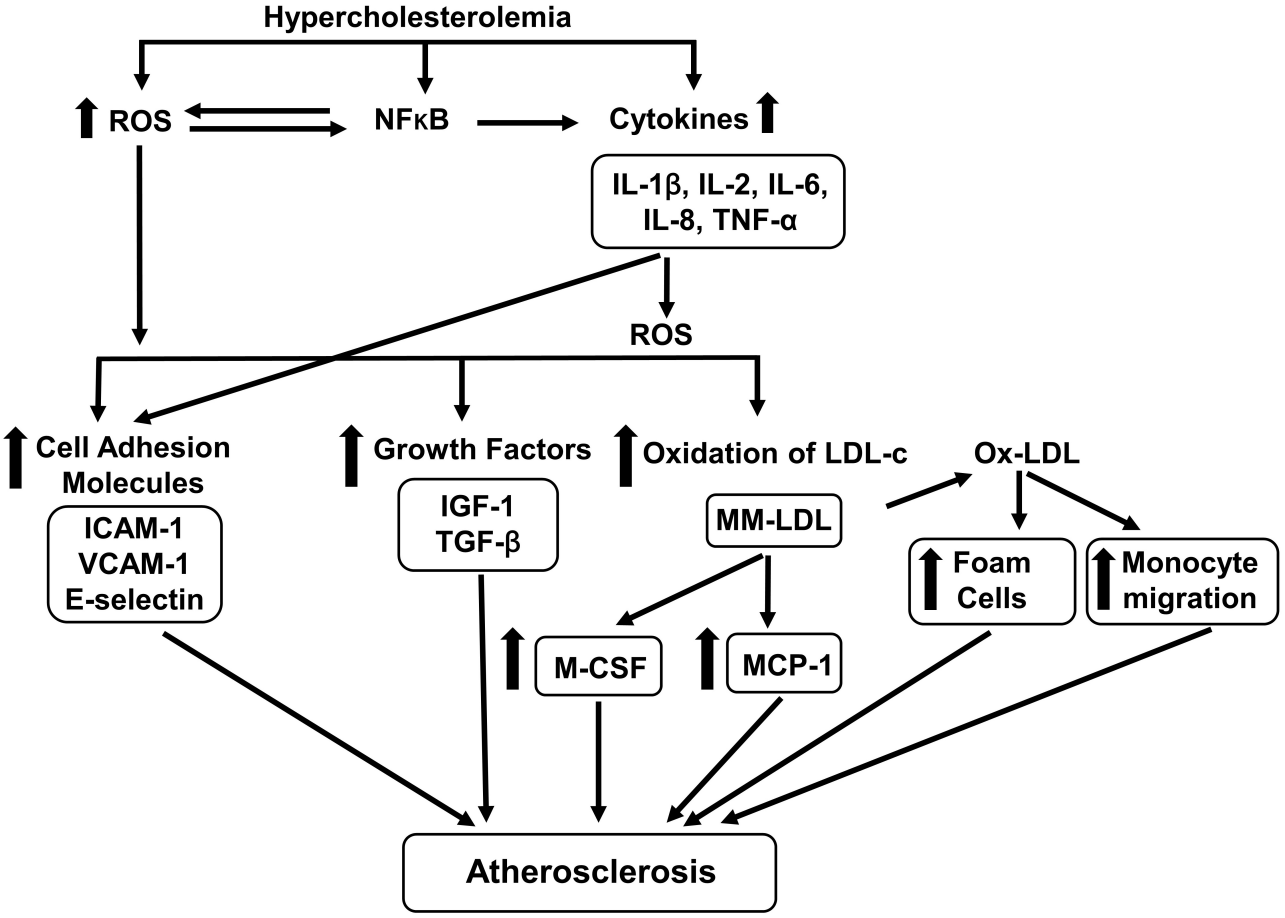
**Effects of hypercholesterolemia on atherogenic biomolecules**. Hypercholesterolemia increases the generation of ROS (reactive oxygen species) and cytokines [interleukin (IL)-1, IL-2, IOl-6, IL-8, tumor necrosis factor-alpha (TNF-α)], and activates nuclear factor-kappa B (NF-κB). Cytokines generate ROS and increase the expression and release of cell adhesion molecules [intercellular adhesion molecule-1 (ICAM-1), vascular cell adhesion molecule-1 (VCAM-1), E-selectin]. ROS increase the expression and release of cell adhesion molecules, growth factors [insulin-like growth factor-1 (IGF-1), transforming growth factor-beta (TGF-β)], and increases oxidation of low-density lipoprotein cholesterol (LDL-C) to form minimally modified LDL (MM-LDL) which is further oxidized to form maximally oxidized-LDL (OX-LDL). MM-LDL produces monocyte chemoattractant protein-1 (MCP-1) and monocyte colony stimulating factor (M-CSF) from endothelial cells. OX-LDL assist in migration of monocytes in subendothelial space and formation of foam cells. All the above biomolecules are involved in the development of atherosclerosis. ⇆, rightward and leftward arrow; ↑, increase.

Atherogenic biomolecules are defined as the biomolecules which are involved in the induction of atherosclerosis. This section describes the hypercholesterolemia-induced production of atherogenic biomolecules (Fig. 1).

### 2.1 Hypercholesterolemia-Induced Sources of ROS

There are various sources of hypercholesterolemia-induced increases in ROS. The content of cholesterol in platelets, polymorphonuclear leucocytes (PMNLs), endothelial cells, smooth muscle cells and monocytes are elevated by hypercholesterolemia [28, 29, 30]. Thrombin, histamine, and adenosine diphosphate (ADP) are released by cholesterol-rich platelets [31, 32]. Phospholipase $A_{2}$ is activated by histamine and ADP [33] which act on membrane phospholipids to release arachidonic acid [34]. Increases in the intracellular ${\mathrm{Ca}}^{2+}$ concentration [35] that occur in hypercholesterolemia [36] would also increase the phospholipase $A_{2}$ activity. The formation of arachidonic acid is enhanced by activated phospholipase $A_{2}$ and hence an increase in the synthesis of prostaglandins and leukotrienes in various cells. The intermediate steps in the biosynthesis of prostaglandins [37] and leukotrienes [38] from arachidonic acid generate ROS. Leukotriene B4 (LTB4) is formed during the metabolism of arachidonic acid by leukocytes. Hypercholesterolemia activates complements component C3 and C5 (C3a and C5a) [39]. The synthesis and release of platelet-activating factor (PAF) are elevated by hypercholesterolemia [35]. Platelet-activating factor increases the formation and release of interleukin-1 (IL-1) [40], and tumor necrosis factor-alpha (TNF-α) [41]. Platelet-activating factor [42], LTB4 [43], C3a and C5a [44], interleukin-1 [45] and TNF-α [46] stimulate PMNLs to generate ROS. Hypercholesterolemia accelerates the production of ROS in endothelial cells through activation of nicotinamide adenine dinucleotide phosphate (NADPH)-oxidase [47, 48]. NADPH-oxidase is the most important modulator of ROS in endothelial cells. The serum levels of CRP in insulin-sensitive subjects are elevated by hyperlipidemia [49]. Prasad [8] has reported that CRP increases the generation of ROS from white blood cells.

### 2.2 Effects of Hypercholesterolemia on Antioxidants 

Reduction in antioxidants would also elevate the serum levels of ROS. Superoxide dismutase (SOD), catalase and glutathione peroxidase (GSH-Px) are enzymatic antioxidants. Superoxide dismutase metabolizes superoxide anion to hydrogen peroxide ($H_{2}$$O_{2}$) and oxygen, while catalase metabolizes $H_{2}$$O_{2}$ to $H_{2}$O + $O_{2}$. GSH-Px metabolizes $H_{2}$$O_{2}$ to water and oxygen. This suggests that the Superoxide anion (oxygen radical) becomes inactive with antioxidant enzymes. Serum levels of SOD and GSH-Px have been reported to be markedly reduced, while catalase activity was elevated in hypercholesterolemic rabbits as compared to control [50]. Vitamin E, an antioxidant, produced an increment in the serum levels SOD and GSH-Px activity without a change in the catalase activity [50]. The activity of aortic SOD, Catalase and GSH-Px were significantly augmented in hypercholesterolemic rabbits [50].

### 2.3 Role of ROS in the Generation of Biomolecules for Development of Atherosclerosis

ROS have numerous functions in the development of atherosclerosis. It activates nuclear factor-kappa-B (NF-κB) [51] which in turn activates pro-inflammatory genes of various cytokines such as, interleukin (IL)-1, IL-2, IL-6, IL-8 and TNF-α and interferon-γ (IFN-γ) [40, 41]. IL-1 and TNF-α stimulate PMNLs to generate ROS [40, 41, 51, 52, 53]. NF-κB is a key factor in regulation of NADPH-oxidase expression and function [54]. ROS elevate the expression of intercellular adhesion molecule-1 (ICAM-1) [55, 56] and vascular cell adhesion molecule-1 (VCAM-1) [57, 58] in endothelial cells. Expression of E-selectin in the human endothelial cell is increased with ROS [59]. The expression of cell adhesion molecules (CAM) is elevated by cytokines [60]. Leukocytes adhesion to endothelial cells is the early step in the development of atherosclerosis [61]. ROS are implicated in the growth, proliferation, and differentiation of vascular smooth muscle cells [62, 63, 64]. Insulin-like growth factor-1 (IGF-1) plays a critical role in the growth of vascular smooth muscle cells [65]. ROS increase the formation of IGF-1 in vascular smooth muscle cells and play an important role in the growth of vascular smooth muscle cells [66]. Transforming growth factor (TGF-β) modulates vascular development and remodeling by cell differentiation, proliferation, migration and extracellular matrix formation [67]. ROS activate TGF-β’s which mediate numerous TGF-β fibrogenic effects [68].

Oxidation of LDL-C by ROS has numerous functions in the development of atherosclerosis [68, 69, 70, 71, 72]. LDL-C is mildly oxidized to form minimally modified LDL (MM-LDL) which is further oxidized to form maximally oxidized LDL (OX-LDL). MM-LDL activates smooth muscle cells and endothelial cells to produce monocyte chemoattractant protein-1 (MCP-1) which is involved in the migration of monocytes (leukocytes) from endothelial surface to subendothelial space. Monocytes possess LDL receptors which combine with native LDL, but the amount of native LDL is not enough to form foam cells. MM-LDL stimulates endothelial cells to generate monocyte colony-stimulating factor (MC-SF) which triggers monocyte differentiation into macrophages that develop receptor for OX-LDL. OX-LDL is taken up by differentiated macrophages to form foam cells. An overview on the formation of OX-LDL and its role in the development of atherosclerosis have been reported by Poznyak *et al*. [73]. Parthasarathy *et al*. [70] have reported that OX-LDL is present in the circulating blood. LDL oxidation takes place in the vascular wall [73]. Hashimoto *et al*. [74] have reported that transmigration of monocytes into subendothelial space is assisted by OX-LDL directly through a change in the endothelial junction. Other investigators [75] have reported that OX-LDL assists in the recruitment of monocytes through interaction of platelet with monocytes and endothelial cells. Macrophages are involved in the generation of numerous growth-regulating factors [76]. Plasma LDL has been shown to have a positive correlation with ROS release by mononuclear leucocytes (MNLs) and polymorphonuclear leukocytes (PMNLs) [77].

Triglycerides (TG) enhance the generation of ROS and secretion of TGF-β and IL-β [78, 79]. Araujo *et al*. [77] have reported that plasma triglycerides were positively correlated with the release of ROS by MNLs and PMNLs. Triglycerides increase the expression of cytokines (IL-1, IL-6, IL-8, TNF-α) [80] and adhesion molecules (ICAM-1, VCAM-1) [81].

HDL-C has antiatherogenic properties. Plasma HDL-C has a negative correlation with ROS release by resting MNLs and PMNLs [77]. It has antioxidant activity [82] and has inhibitory effects on LDL oxidation [83]. HDL-C reduces the expression of MCP-1 [84] and prevents the CRP-induced upregulation of proinflammatory adhesion molecules [85].

## 3. Mechanism of Hypercholesterolemia-Induced Atherosclerosis 

**Fig. 2.**
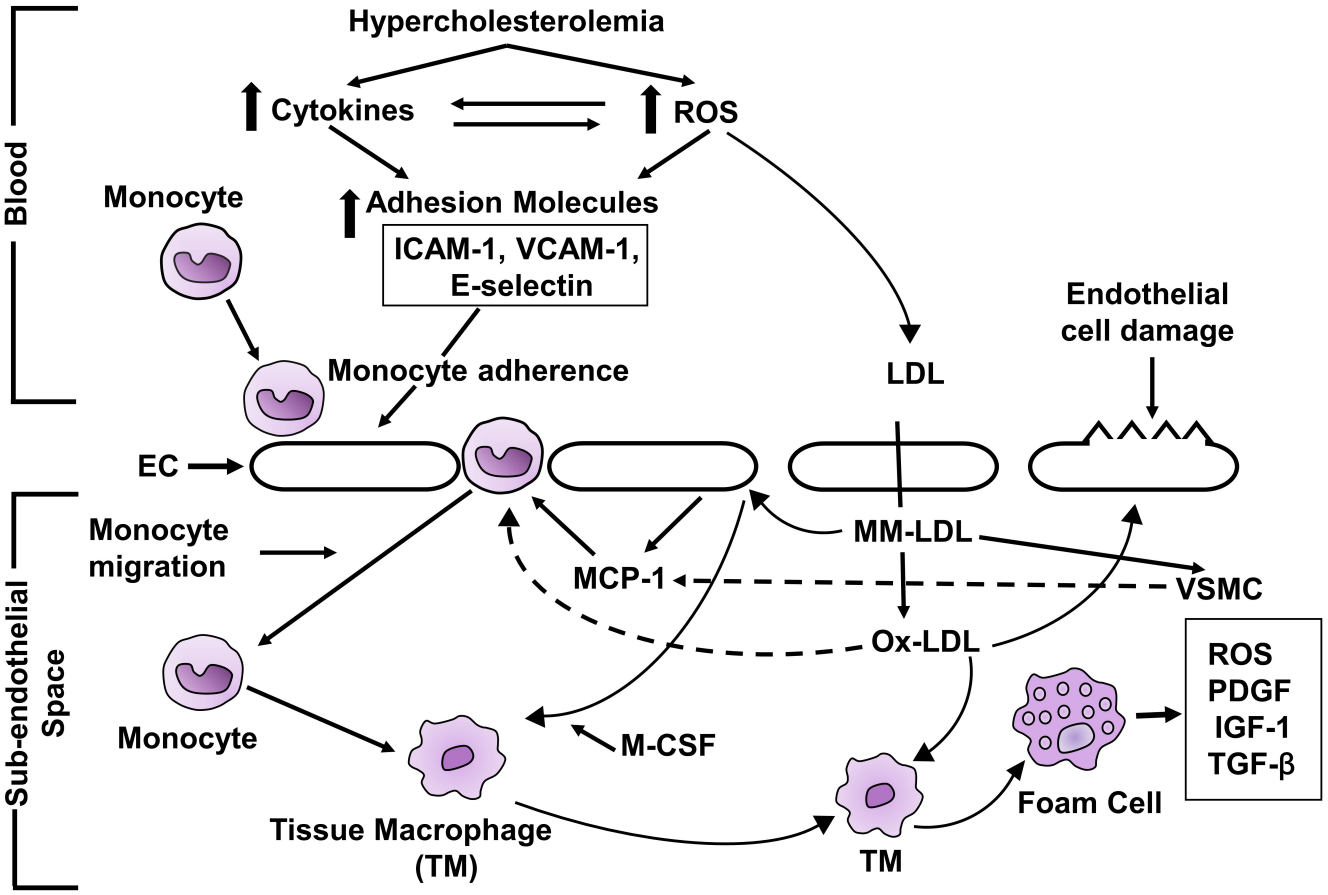
**Schematic diagram of mechanism of hypercholesterolemia-induced atherosclerosis**. ROS, reactive oxygen species; ICAM-1, intercellular adhesion molecule-1; VCAM-1, vascular cell adhesion molecule-1; EC, endothelial cell; LDL, low-density lipoprotein; MM-LDL, minimally modified LDL; OX-LDL, maximally oxidized LDL; MCP-1, monocyte chemoattractant protein; VSMC, vascular smooth muscle cell; MC-SF, monocyte colony stimulating factor; TYM, tissue macrophage; PDGF, platelet-derived growth factor; IGF-1, insulin-like growth factor-1; TGF-β, and transforming growth factor-β. ↑, increase; ⇆ , rightward and leftward arrow.

Hypercholesterolemia-induced atherosclerosis is based on the oxidative hypothesis of atherosclerosis which has been accepted universally [71, 72, 76, 86]. The proposed mechanism of atherosclerosis produced by hypercholesterolemia is depicted in Fig. 2. Hypercholesterolemia augments the production of ROS [37, 38, 42, 43, 44, 45, 46] and cytokines [40, 41] which increase the expression of CAM [55, 56, 57, 58]. CAM [55, 56, 57, 58] in endothelial cells. The early step in the development of atherosclerosis is adherence of monocytes to endothelial cells [61] and which is achieved through CAM. CAM is involved in the rolling and adhesion of monocytes to the endothelial cells. Monocyte then transmigrates into subendothelial space [87]. MM-LDL produce monocyte chemoattractant protein-1 (MCP-1) in endothelial cells and vascular smooth muscle cells [88]. The migration of monocytes to the subendothelial space is assisted by MCP-1 [89]. OX-LDL increases the expression of cell adhesion molecules [90]. OX-LDL directly enhances the migration of monocytes to subendothelial space. Immigrating monocytes into the subendothelial space have LDL receptor but the rate of uptake of native LDL is not enough to produce foam cells [91]. MM-LDL stimulates endothelial cells to express MC-SF [92] that enhances the monocyte differentiation to form tissue macrophages which develop receptors for OX-LDL [92]. OX-LDL is a ligand for scavenger receptors which are expressed in tissue macrophages [93]. OX-LDL is taken up by tissue macrophage to form foam cells. Foam cells are involved in formation of numerous growth factors which enhance vascular smooth muscle cell proliferation and migration and fibrous tissue synthesis which helps in the development and progression of atherosclerosis. There is a development of fatty streaks in full-fledged atherosclerosis.

## 4. Evidence for the Role of Hypercholesterolemia-Induced ROS in the Development of Atherosclerosis

As described above, hypercholesterolemia generates ROS. The question arises if hypercholesterolemia-induced ROS induces atherosclerosis. This section describes the increases in the levels of ROS and the indirect measures of ROS in hypercholesterolemic atherosclerosis. Indirect measures of ROS include lipid peroxidation products, malondialdehyde (MDA) [94, 95], aortic tissue chemiluminescence (AO-CL) [96], a polymorphonuclear leukocyte chemiluminescence (PMNL-CL) [97] and white blood cell chemiluminescence (WBC-CL) [97]. AO-CL is a measure of antioxidant reserve [96]. An increase in AO-CL suggests a decrease in the antioxidant reserve and vice-versa. Luminol-dependent chemiluminescence is a highly sensitive method for measurement of ROS generated by PMNLs and WBCs [97].

Hypercholesterolemic atherosclerosis was associated with increases in the serum [20, 97, 98, 99, 100, 101] and aortic MDA [19, 96, 97], PMNL-CL [96], WBC-CL [96, 98, 101] and aortic-CL [96, 97]. However, the aortic-CL has been observed to be reduced in certain studies [98, 99, 100, 101, 102]. Aortic-CL is a measure of both oxidative stress and antioxidant reserve in the tissue [103]. If hypercholesterolemia increases the indirect measure of ROS and produces atherosclerosis, then lowering serum levels of cholesterol would be associated with reduction in the extent of atherosclerosis and the levels of both direct and the indirect measure of ROS. We describe the agents which have both antioxidant and hypolipidemic effects on hypercholesterolemic atherosclerosis and ROS. Secoisolariciresinol diglucoside (SDG) a product of flaxseed reduced the serum levels of cholesterol and this reduction was associated with a reduction in the extent of hypercholesterolemic atherosclerosis, aortic MDA and aortic-CL [104]. Flax lignin complex, a byproduct of flaxseed reduced hypercholesterolemic atherosclerosis by 30%, and this effect was associated with a lowering of serum levels of cholesterol by 20%, serum MDA by 35% and aortic MDA by 58% in rabbits [99]. It is to note that both SDG and flax lignan complex have antioxidant activity [99, 105, 106]. Probucol, an antioxidant and cholesterol-lowering agent [107] decreased the extent of hypercholesterolemic atherosclerosis, and aortic tissue MDA, but had no effects on aortic-CL [96].

We now discuss the effects of antioxidants on hypercholesterolemic atherosclerosis and ROS. Since ROS is implicated in the formation of atherosclerosis, the antioxidants would reduce the evolution of hypercholesterolemic atherosclerosis and associated indirect measures of ROS. Vitamin E, an antioxidant [108], reduced hypercholesterolemic atherosclerosis and this was associated with a decrease in serum and aortic MDA but had no effect on serum cholesterol [20].

Sources of hypercholesterolemia-induced ROS include the synthesis of prostaglandins and leukotrienes [37, 38], activated complements [39, 44], PAF [42], and cytokines [45, 46]. Hence inhibitors of the enzyme of synthesis of prostaglandin and leukotrienes, PAF, cytokines and activated compliments would decrease the formation of hypercholesterolemic atherosclerosis and ROS levels. Inhibitors of cyclooxygenase which is involved in the synthesis of prostaglandin and leukotrienes such as aspirin [109], and indomethacin [110] were used in the prevention of hypercholesterolemic atherosclerosis and reduction of ROS. Aspirin did not affect the serum levels of cholesterol in rabbits with hypercholesterolemia but reduced atherosclerosis by 47% and this effect was associated with lowering of serum and aortic tissue MDA, release of ROS from WBC-CL, and aortic-CL [101]. Indomethacin decreased the extent of hypercholesterolemic atherosclerosis by 46% and this effect was associated with a decrease in aortic MDA and antioxidant reserve, but no change in the serum cholesterol, and WBC-CL [111]. Pentoxifylline an inhibitor of cytokines [112], and PAF [113, 114] had no effect on serum cholesterol but the extent of hypercholesterolemia-induced atherosclerosis was lowered by 38% and this effect was associated with a reduction in serum and aortic tissue MDA, and normalization of aortic-CL [115].

## 5. Serum/Plasma/Tissue Levels of Atherogenic Biomolecules in Hypercholesterolemia 

Are the atherogenic biomolecules such as serum/plasma/tissue levels of ROS, NADPH-oxidase, NF-κB, CAM, cytokines, MCP-1, GM-CSF, PAF, LTB4, activated complements, IGF-1, and TGF-1 elevated in hypercholesterolemia? One would expect these atherogenic biomolecules to be elevated in hypercholesterolemia.

The increases in the serum/tissiue levels of ROS in hypercholesterolemic rabbits have been described in detail in section 4 of this review. Hypercholesterolemia increases the activity of the oxidant producing enzyme system, NADPH-oxidase [116], and xanthine oxidase [117]. Hypercholesterolemia activates NF-κB [118]. Circulating NF-κB is elevated in familial hypercholesterolemia [119]. Hypercholesterolemia increases the soluble cell adhesion molecules (sICAM-1, sVCASM-1, sE-selectin) [120, 121, 122]. The serum levels of IL-6, IL-8, IL-12, TNF-α and IFN-γ increased, while that of IL-4 and IL-10 decreased in hypercholesterolemia [123, 124, 125]. Hypercholesterolemia increases the levels of circulating MCP-1 [123]. The serum levels of GM-CSF are elevated in hypercholesterolemic patients [126]. Plasma levels of PAF have been reported to rise in hypercholesterolemic patients [127]. Plasma levels of LTB4, which promotes atherosclerosis [102], are elevated in hypercholesterolemic rats [128]. Activated C3 is elevated in hypercholesterolemic apo-E-null mice and patients with familial hypercholesterolemia [129]. In summary, the atherogenic biomolecules are elevated in hypercholesterolemic subjects.

## 6. Involvement of AGE and Its Receptors in Hypercholesterolemic Atherosclerosis

AGEs are heterogenous groups of irreversible adducts produced from the nonenzymatic interaction of amino groups of protein, lipids, and nucleic acids with reducing sugars such as glucose, fructose, and glyceraldehyde [130, 131]. Receptors for AGE include RAGE, sRAGE, esRAGE, and cRAGE (cleaved RAGE). RAGE is bound to the cell membrane, while sRAGE, esRAGE, and cRAGE circulate in the blood. RAGE has two isoforms, esRAGE and cRAGE. cRAGE is cleaved from RAGE by proteolytic enzymes [132] and esRAGE is produced from alternate mRNA splicing of full-length RAGE [133]. sRAGE contains both cRAGE and esRAGE. sRAGE, esRAGE, and cRAGE lack the cytosolic and transmembrane domain and circulate in the blood. Interaction between AGE with RAGE produces atherogenic biomolecules [23, 134]. The binding of sRAGE, cRAGE and esRAGE with AGE does not activate intracellular signaling and does not produce atherogenic biomolecules. There is a competition between RAGE and sRAGE for binding with AGE [135]. Thus, sRAGE and esRAGE have protective effects against adverse effects of interaction of AGE with RAGE. AGE-RAGE stress, defined as the ratio of AGE/sRAGE has been coined by Prasad and Mishra [136], A high ratio of AGE/sRAGE indicates the presence and progression of atherosclerosis.

The serum levels of AGE and AGE/sRAGE were higher, while the sRAGE levels were lower in hypercholesterolemic subjects than normocholesterolemic subjects [137]. The above investigators also reported that there was a positive correlation between serum cholesterol levels and the levels of AGE and AGE/sRAGE, and a negative correlation between serum cholesterol and sRAGE. Santilli *et al*. [138] have also reported that hypercholesterolemic subjects had lower serum levels of sRAGE than normocholesterolemic subjects. Hypercholesterolemia-induced AGE would interact with RAGE to generate ROS [139], which would activate NF-κB [51] and has been discussed in detail in the section on “Role of ROS in the development of atherosclerosis” of this paper. The mechanism of AGE-RAGE stress in the formation of atherosclerosis has been described in detail elsewhere [23, 134]. The following section provides the evidence of the implication of AGE, RAGE and sRAGE in the development of atherosclerosis.

The levels of AGE and RAGE were elevated in the wall of the carotid artery of Zucker diabetic rats, and these levels were further elevated in the balloon-injured carotid artery of these rats [140]. These authors also reported that sRAGE administration before and for 21 days post-balloon injury reduced the neointimal hyperplasia in the carotid artery. De-endothelialization of the carotid artery in wild type mice has been shown to elevate the expression of RAGE in injured arteries [141]. They also observed that use of sRAGE reduced neointimal hyperplasia in these mice. Wendt *et al*. [27] have shown that diabetes-accelerated atherosclerosis in apo-E deficient mice had increased expression of VCAM-1 in the aorta, and that sRAGE administration significantly reduced the atherosclerotic lesion in the aorta. Administration of sRAGE completely suppressed the accelerated and advanced atherosclerosis in apo-E deficient mice [142]. Serum levels of sRAGE were reduced in Non-ST-segment elevated myocardial infarction [25]. Serum levels of sRAGE were reduced in patients with restenosis following percutaneous coronary intervention (PCI) [26]. Low pre-PCI sRAGE levels in serum have been reported to be a predictor of post-PCI restenosis in NSTEMI patients [26]. AGE-RAGE stress has been reported to play a role in the development of coronary artery disease [134, 143] and carotid artery stenosis [144].

## 7. Role of CRP in Hypercholesterolemic Atherosclerosis

A hypercholesterolemic diet increases the serum levels of CRP [49]. CRP can induce atherosclerosis through the generation of ROS [8, 145, 146] activation of NF-κB [147], and increased expression of CAM [148], and MCP-1 [149]. CRP increases the release of MC-SF [150]. CRP has been implicated in the development of CAD, peripheral vascular disease, and post-PCI restenosis [151]. The data suggest that hypercholesterolemia- induced increase in CRP could also be involved in the development of hypercholesterolemic atherosclerosis through generation of numerous atherogenic biomolecules.

## 8. Perspectives 

Hypercholesterolemia increases the production of ROS which sets the stage for the production of other atherogenic biomolecules [27, 28, 29, 30, 31, 32, 33, 34, 35, 36, 37, 38, 39, 40, 41, 42, 43, 44, 45, 46, 47, 48] leading to the formation of atherosclerosis. Reduction in antioxidant enzymes by high blood cholesterol would also elevate the ROS levels [50]. Hypercholesterolemia-induced atherosclerosis is associated with increases in the serum/plasma/tissue levels of direct and indirect measures of ROS [19, 20, 96, 97, 98, 99, 100, 101, 104]. Blockade of the ROS with antioxidant (vitamin E) [20], hypolipidemic and antioxidant agents (SDG [104], flax lignan complex [99], and probucol [96]), cyclooxygenase inhibitors (aspirin) [101] and indomethacin [111], and inhibitors of cytokines and PAF (pentoxifylline [115]) decreased the development of hypercholesterolemic atherosclerosis and amount of ROS. The above data indicate that there is an association between hypercholesterolemic atherosclerosis and ROS, while lowering the serum cholesterol and blockade of sources ROS reduces the extent of atherosclerosis and ROS. It is to note that hypercholesterolemia elevates the serum levels of AGE [137] and AGE/sRAGE [137], and lowers the serum levels of sRAGE [135, 136]. An increase in AGE and AGE/sRAGE, and a decrease in sRAGE in the serum have been implicated in the development of atherosclerosis [23, 134, 137]. Hypercholesterolemia has been reported to elevate the serum levels of CRP in human subjects [49]. A rise in C-reactive protein increases the serum levels of atherogenic biomolecules [146, 147, 148, 149, 150] and induces development of atherosclerosis [151]. It is surprising that there are limited publications on the effects of hypercholesterolemia on C-reactive protein and AGE-RAGE axis. Hypercholesterolemia increases the production of AGE, CRP, and ROS, and decreases the production of sRAGE all of which are implicated in the formation of atherosclerosis. Lowering of AGE and C-reactive protein, raising of sRAGE, and use of antioxidants may be considered as an adjunct therapy besides lipid lowering agents for the treatment of hypercholesterolemia.

## 9. Conclusions

Hypercholesterolemia induces atherosclerosis through increases in the atherogenic biomolecules (ROS, NADPH-oxidase, NF-κB, CAM, MCP-1, GM-CSF, cytokines, MM-LDL, OX-LDL and growth factors). The initiating atherogenic biomolecule is ROS. Lipid-lowering agents, antioxidants, and the agents that block the sources of atherogenic biomolecules would reduce the development of hypercholesterolemic atherosclerosis. Hypercholesterolemia could also produce atherosclerosis through increases in AGE, AGE/sRAGE and CRP, and decreases in the levels of sRAGE.
